# Reflections on Epistemic Injustice to Advance Person-Centred Care Through the Experiences of Persons with Chronic Pain

**DOI:** 10.1007/s11673-025-10457-0

**Published:** 2025-07-29

**Authors:** Felix Gabathuler, Kristina Würth, Martina Hodel, Andrea Glässel, Nikola Biller-Andorno, Bettina Schwind

**Affiliations:** 1 Institute of Biomedical Ethics and History of Medicine (IBME), University of Zurich, Winterthurerstrasse 30, 8006 Zurich, Switzerland; 2 Institut des Humanités en Médecine (IHM), Université de Lausanne, Avenue de Provence 82, 1007 Lausanne, Switzerland

**Keywords:** Chronic pain, Person-centred care, Bioethics, Medical ethics, Physician–patient relations, DIPEx

## Abstract

Rationale: Persons with chronic pain report that their voices are marginalized in healthcare, despite efforts to achieve person-centred care. Aims and Objectives: This study aims to explore the healthcare experiences of persons with chronic pain through the lens of epistemic injustice to advance person-centred care. Method: A secondary analysis of cross-sectional interviews with twenty German-speaking Swiss participants, originally collected as part of the DIPEx Switzerland project, was conducted. Data were examined using thematic analysis. Results: Results revealed two overarching themes. Under *Epistemic Challenges*, participants felt dismissed, misunderstood, or relegated to passive roles by a system privileging quantifiable measures over subjective experiences. This overreliance on objective data fosters epistemic injustice by discounting patient testimonies and perpetuating systemic inadequacies. Under *Epistemic Opportunities*, participants reported more effective knowledge exchange when their expertise was acknowledged, empathy was shown, and professionals recognized their own limitations. Conclusions: Findings underscore the need to balance objective assessments with patients’ subjective perspectives, recognizing persons with chronic pains as legitimate collaborators. By integrating their lived expertise, healthcare systems may mitigate epistemic injustices and provide more empathetic, effective care.

## Introduction

Chronic pain, which is defined as pain lasting for more than three months, is recognized as a major public health challenge, affecting more than 30 per cent of the global population (Cohen et al. 2021; Raffaeli et al. 2021). In Switzerland, 11.6–16 per cent of adults are estimated to experience moderate to severe chronic pain (Breivik et al. 2006; Dirupo et al. 2024). Despite its prevalence, care for persons with chronic pains (chronic pain persons, CPP) remains in need of improvement (Cohen et al. 2021). Although initiatives in Switzerland—such as Project PrePac (n.d.) and efforts by the Swiss Pain Society (n.d.)—aim to improve care for CPP, the country’s healthcare system is considered fragmented, lacking a unified approach to chronic disease management (Federal Office of Public Health 2019; Nolte et. al 2014). Its structures are recognized as overly geared toward acute care and do not sufficiently address the needs of the growing number of people living with chronic illnesses (Federal Office of Public Health 2019).

Person-centred care is considered key to the clinical management of long-term conditions, such as chronic pain, with improved health outcomes cited as a key benefit (Légaré et al. 2018; NICE 2021; Tattersall 2002). It recognizes patients as experts in their experiences, preferences, and needs, striving for personalized care through shared decision-making and respectful therapeutic relationships (Castro et al. 2016; Håkansson Eklund et al. 2019). While “patient-centred” and “client-centred” are largely synonymous, the term “person-centredness” is increasingly preferred for emphasizing that patients are individuals beyond their medical conditions (Håkansson Eklund et al. 2019).

Despite efforts to implement person-centred care, fully integrating individuals with long-term conditions—including chronic pain—into clinical decision-making remains a challenge (Ahmad et al. 2014; Entwistle et al. 2018; Légaré et al. 2018; Pieterse and Finset 2019). CPP frequently report feeling dismissed, stigmatized, and having their testimonies overlooked by healthcare professionals (HCP) (MacNeela et al. 2015; Nicola et al. 2021; IPRCC 2019; Wallace et al. 2021). Correspondingly, policy and research call for addressing this disregard of patient expertise, highlighting the need to tailor care to each individual’s unique circumstances (Baker et al. 2023; Institute of Medicine (US) Committee on Advancing Pain Research, Care, and Education 2011; NICE 2021). Patient expertise may be defined as “experiential knowledge gained from personally managing day-to-day experiences of illness” (Hartzler and Pratt 2011, 22). The dismissal and devaluation of experienced-based knowledge in clinical practice may not only inhibit person-centred care (Rogers et al. 2005) but also potentially lead to epistemic injustice (Carel and Kidd 2014; Kidd and Carel 2017).

Epistemic injustice, a concept introduced by Miranda Fricker, occurs when individuals are wronged “specifically in their capacity as knowers” (Fricker 2007, 20). Fricker differentiated between two forms of epistemic injustice: First, testimonial injustice arises when a speaker’s credibility is doubted due to the listener’s biases; Second, hermeneutical injustice emerges when there is a lack of shared or recognized terminology to describe and validate the speaker’s experiences. Both forms of injustice can leave patients at a systemic disadvantage, undermining their capacity to be recognized as knowers. For example, minority patients with mental health concerns may face scepticism (testimonial injustice), or cultural differences in symptom expression may lead to misunderstanding or misdiagnosis (hermeneutical injustice). Other scholars have built upon Fricker’s framework by introducing additional types of epistemic injustices that extend beyond her initial conceptualization (Hookway 2010; Dotson 2011).

Research on epistemic injustice in healthcare has increased in recent years, largely focusing on the fields of ethics, philosophy, mental health, and psychiatry (see for examples Kidd’s (2024) web bibliography). However, despite an extensive literature search, we found only limited empirical studies on epistemic injustice in relation to chronic pain care: Buchman et al. (2017) apply a bioethical lens to highlight the marginalization of CPP voices. By “bioethical,” we refer to the systematic study and evaluation of the moral, ethical, and societal dimensions of medicine (Gordon, n.d.). Tosas (2021) also employs a bioethical approach to analyse medical humanities literature on the downgrading of CPP credibility, stressing the need for research into the moral and clinical implications of believing or disbelieving CPP. In a Spanish qualitative study (García-Rodríguez et al. 2023) use a sociological perspective to examine how systemic factors contribute to epistemic injustice experienced by CPP in workplace, family, and healthcare settings. They call for further empirical research to better understand the rigidity and challenges within healthcare systems in addressing chronic pain. While these studies address epistemic injustice in chronic pain, they do not fully explore its consequences for person-centred care. However, other research suggests that addressing epistemic injustice could improve person-centred care by evaluating how patient knowledge is—or is not—valued in clinical settings (Bogaert 2021; Dell’Olio et al. 2023; Naldemirci et al. 2021).

The current study aims to investigate whether and how epistemic injustice manifests in the healthcare experiences of CPP, as well as its impact on their perceptions of care quality and involvement in decision-making. Thereby, we seek to identify both barriers and facilitators to implementing genuine person-centred care in chronic pain management and, ultimately, to contribute to more equitable and respectful healthcare practices.

## Methods

### Background

This study is based on a thematic analysis of qualitative interviews conducted within the “Living with Chronic Pain” project. This project is part of DIPEx.ch, which is a member of the international DIPEx network. In this network, qualitative researchers systematically collect and publicize patient experiences, adhering to established research methods (Our Research—DIPEx International 2024).

All team members, except FG who is male, were women. FG and NBA graduated medical school, with FG currently practicing medicine. BS, KW, and AG come from diverse professional backgrounds but specialize in qualitative research. MH is a clinical psychologist. NBA and AG led the design of the DIPEx Chronic Pain project. Interviews were conducted by AG, MH, KW, and BS. FG, BS, and KW contributed mainly to the design and analysis of the current study, while all authors were involved in the process.

### Methodology and Ethical Considerations

We followed COREQ reporting criteria (Tong et al., 2007) and conducted the study in accordance with the Declaration of Helsinki (World Medical Association 2013) and all applicable national regulations. The DIPEx.ch project was reviewed by the ethics review committee of the Canton of Zurich which determined that the project did not fall under the Swiss Human Research Act, granting a national exemption (BASEC No. req-2018–00050). Additionally, the content of the present study was reviewed and approved by the internal review board of the University of Zurich.

### Sampling and Recruitment

Within DIPEx.ch maximum variety sampling to ensure diversity among CPP was employed (Patton 2014). Factors considered included language region in Switzerland (German, French, Italian), age, gender, education, and residence (urban to rural). Eligible participants were adults (aged eighteen and older) who had experienced pain for a minimum of three months. Those with pain under three months, major psychological disabilities, or inadequate language skills were excluded. Participants had no prior relationship with the researchers.

This article analyses twenty interviews conducted with German-speaking participants. Participants were recruited from various sources, including university hospitals in Zurich and Basel, chronic pain self-help groups, the Rheumatism League Switzerland, and private medical practices. Informed consent was obtained in written form from all participants prior to their participation.

### Data Collection

This study represents a secondary analysis carried out using the theoretical framework of epistemic injustice, which was not specifically considered in the original DIPEx interviews. The data were collected from April 2018 to September 2021 through semi-structured interviews that began with an open-ended narrative component. They included focusing on six main areas: 1. Onset, 2. Experiences, 3. Management, 4. Daily life impact, 5. Healthcare interactions, and 6. Personal insights regarding chronic pain.

Interviews were conducted either in person or online (via Teams or Zoom) with only the interviewer and the participant present and lasted between 60 to 120 min. All sessions were recorded, in either video or audio format based on consent, then transcribed and pseudo-anonymized.

### Data Analysis

We employed thematic analysis with a mixed inductive/deductive approach (Braun and Clarke 2006, Clarke and Braun 2014; Byrne 2022) using the MAXQDA Standard 2020 software. Initial themes related to CPP experiences in healthcare emerged from the data and were then compared to aspects of epistemic injustice. We followed the six steps in thematic analysis as formulated by Braun and Clarke (2006) and Clarke and Braun (2014): 1. Familiarization, 2. Generate initial codes (inductively), 3. Preliminary theme identification (inductively), 4. Theme review (reviewing preliminary themes with aspects of epistemic injustice), 5. Theme definition, and 6. Producing the report.

Interview excerpts were translated from German to English and selectively paraphrased for clarity, with care taken to preserve the intended meaning. All changes were documented. Participants did not provide feedback on the findings.

## Results

### Participants Characteristics

Participants, aged thirty-eight to seventy-three (one person did not provide the age), suffered from chronic pain conditions such as fibromyalgia, rheumatoid arthritis, migraines, and pain from severe injuries or genetic disease. The group included eleven women and nine men. Nine had completed tertiary education, nine secondary education, and two other forms of education. Twelve were working and eight were not working, with two being retired and three receiving invalidity insurance. They are referred to as P1-20 herein.

### Results from Analysis

Two themes resulted from the analysis: 1. Epistemic Challenges, and 2. Epistemic Opportunities. Each theme comprises two sub-themes (see Table 1).

**Table 1:** Overview of themes and sub-themes

Themes	Subthemes
Epistemic Challenges	- Feeling Discredited
	- Feelings of Systemic Shortcomings in Healthcare
Epistemic Opportunities	- Feeling Recognized as Persons and Contributors to Clinical Processes
	- Feeling Treated Respectfully

### Theme: Epistemic Challenges

#### Sub-Theme 1: Feeling Discredited

Within this sub-theme, participants described experiences where they felt their testimonies were doubted and their pain experiences were not taken seriously. P17 reflected, “The way one is treated, it’s unbelievable … nobody took me seriously there.” P20 echoed this sentiment, stating, “Sometimes I do feel … one is not really taken seriously.” These quotes underscore participants sense of feeling that their testimonies were considered untrustworthy, describing it as an “unbelievable” experience.

Participants described their pain as subjective, invisible, or difficult to measure when explaining why they felt dismissed. P14 shared, “She was one of those doctors who, if she couldn’t find a cause, assumed it was nothing.” Similarly, P15 noted: “Many doctors … feel it doesn’t exist, it’s pure imagination.” These comments reflect participants’ perception that without confirmatory medical tests, their pain is deemed illegitimate, highlighting the authority participants perceive HCP to hold in validating or dismissing their experiences.

The structural authority as experienced by participants is further illustrated by P02’s account: “When I once told a doctor of my traumatizing experiences, he pigeonholed me as having psychosomatic issues, as if that was less acceptable. I felt degraded.” Similarly, P14 recalled being told the following: “He said that I was mentally ill and nothing else,” while P15 stated, “I was brushed off as imagining everything and that it’s all just psychological.” These accounts reflect the participants’ belief that HCP can wield a diagnostic authority that reduces chronic pain to a psychological problem. Participants felt that HCP attributing their pain solely to psychological causes was dismissive and counterproductive, providing no support for managing their pain.

#### Sub-Theme 2: Feelings of Systemic Shortcomings in Healthcare

Whereas Sub-Theme 1 emphasized feelings of interpersonal mistrust and disbelief, this sub-theme focuses on the broader healthcare system that participants felt was ill-suited to their needs. They felt HCP struggled to grasp the complexity of their pain experiences. P02 explained, “And those are strange symptoms … they don’t fit the picture … They couldn’t fully understand them.” Despite considerable efforts to discover an effective way to manage their pain—consulting numerous HCP and attempting various therapies—participants like P19 reported that healthcare was unable to help them: “All these things have never really helped me … I never made any significant progress.” Similarly, P04 observed a sense of helplessness among HCP, stating, “The doctors are helpless … we don’t have a clue.” These statements reflect the uncertainty, helplessness, and lack of understanding participants expressed sometimes to perceive in their HCP.

Beyond these practical challenges, several participants also voiced broader concerns about the healthcare system’s capacity to investigate and address chronic pain. They reported that from their perspective economic factors and structural constraints can limit diagnostic thoroughness. P05 illustrated this scepticism: “There has NEVER been a doctor who really investigated the underlying cause of my pain. … there is no intention to address my issue … maybe it is medically irrelevant, or there’s just no money to be made …”.

Others reasoned about the specialization within healthcare which they described as a barrier to comprehensive or holistic treatment. For instance, P03 remarked: “The more specialized people are, the harder it probably is [to perceive people]. … the psychological side is also important.” We assume that by “specialization,” the participant refers to the increasing focus of HCP on narrow subfields of medicine. These observations highlight a perceived lack of integrated approaches to chronic pain management and indicate that participants view system-level pressures—such as economic constraints and narrow specialization of HCP—as barriers to the holistic care they desire.

### Theme: Epistemic Opportunities

#### Sub-Theme 1: Feeling Recognized as Persons and Contributors to Clinical Processes

Participants felt more engaged in their care when HCP acknowledged their expertise and perspectives. P06 shared, “I like doctors … who say: ‘You are the expert of your body; we listen to you, are concerned about how you feel … we are a team.’ So, I like people who acknowledge my expertise as a patient …” This statement underscores how a collaborative approach fostered a sense of partnership. P02 reinforced this perspective, observing that some HCP had come to recognize, “That patients are people who know best about their personal history.”

Feeling recognized as individuals beyond their pain and empowered to contribute to clinical processes appeared crucial in fostering acknowledgment and understanding during clinical consultations. Participants also described how being asked about their goals and wishes encouraged self-reflection and deeper involvement in decision-making. P03 recalled, “This I will never forget … meeting my homeopath for the first time, he asked: ‘What do you want from me?’ … no doctor had ever asked me that before. This made me ponder.” Such experiences stood in contrast to encounters where participants felt their wishes were overlooked.

At the same time, feeling heard and taken seriously meant for participants that they saw HCPs as genuine partners rather than all-knowing authorities. P19 reflected, “That’s an honest attitude. I think that’s better than when he pretends to … know … it’s much more of a discussion, what we want to try … general practitioner as a coach … and not the all-knowing.” Similarly, P01 valued clinicians who acknowledged their limitations, stating, “It’s also nice when you meet physicians who are aware that they do not know everything and openly admit that.”

Additionally, several participants noted that once HCP became aware of their background in the medical field —such as being qualified health scientists—the interaction evolved into a more collaborative partnership. P01 explained, “If one has a medical background … I have often received the feedback … ‘you have got so much knowledge, that’s great.’ You can assess … and know what’s good for you.” Meanwhile, P11 observed: “They put aside more time for me. They talk with me like they’re talking to another physician … They do involve me tremendously in decisions.” From the point of view of these participants, recognition of their medical knowledge enhanced collaboration and mutual respect, leading to more meaningful and equal decision-making processes.

#### Subtheme 2: Feeling Treated Respectfully

This subtheme highlights participants’ emphasis on respect and empathy in clinical relationships. P16 shared, “I think he just very much perceived me with my personality and … was very empathetic towards me. He just knew what was important for me and … supported me. … That was a beautiful experience for me.” P03 similarly described the significance of genuine interaction, stating, “He just properly looks at me first … shakes hands … an interaction is there.”

In these quotes, participants express that respectful care goes beyond acknowledging their expertise or meeting medical needs. Non-verbal behaviours—such as eye contact, a handshake, and a genuine greeting— are emphasized to help convey empathy and validate the patient as a person. For many participants, such interpersonal warmth was reported to be crucial to building trust and fostering a supportive atmosphere in clinical encounters.

## Discussion

The participants described both challenges and opportunities within healthcare settings. They said to have encountered distrust, lack of support, and dismissal of their pain, compounded by their description of systemic inadequacies of the healthcare system, which appeared to not adequately address their needs. Participants described positive encounters with HCP when they felt that their limitations were acknowledged, their role as active contributors was validated, and empathy was expressed through both words and body language. Such reported interactions were apprehended by participants to foster a sense of mutual respect, collaboration, and meaningful involvement in their own care. When participants conveyed their own medical expertise to HCP, they felt the HCP treated them as equals, fostering a more in-depht knowledge exchange.

While objectivity in medicine undeniably bolsters clinical standards (Sackett et al. 1996), we contend that the healthcare system’s heavy reliance on objective data fosters significant epistemic injustice in chronic pain care. By design, medicine prioritizes measurable objective indicators—such as lab results, imaging, and diagnostic tests—to ensure diagnostic uniformity (Kidd and Carel 2017; Wardrope 2015). However, chronic pain rarely yields definitive biomarkers, creating a discrepancy between patients’ lived realities and their medical representation (Buchman et al. 2017; Scarry 1985; Tosas 2021).

In our study, participants described feeling mistrusted and dismissed. When diagnostic tests failed to confirm their pain, they reported their symptoms being reframed as non-existent or psychological. These findings align with previous research indicating that CPP feel marginalized and not taken seriously in clinical encounters (MacNeela et al. 2015; Nicola et al. 2021; Roche and Harmon, 2017). In the absence of objective evidence to substantiate their pain, HCP may doubt or dismiss patients’ experiences (MacNeela et al. 2015; Nicola et al. 2021; Roche and Harmon 2017). This may exemplify epistemic injustice, as subjective accounts that deviate from biomedical norms thereby become systematically devalued (Carel and Kidd, 2014; Kidd and Carel 2017). We argue that latent prejudices—rooted in stereotypes of CPP and implicit doubts about their credibility—further deepen this dismissal, thus constituting a form of testimonial injustice (Buchman et al. 2017; Tosas 2021).

Participants noted that HCP sometimes appeared powerless or unable to fully comprehend their condition, a challenge that may be exacerbated by the intrinsic difficulties of treating chronic pain. Given the limited prospects for pain reduction and modest treatment outcomes (Cohen et al. 2021), it is understandable that participants at times perceived their care as ineffective. Moreover, implicit power dynamics place HCP in a privileged position to determine which knowledge is considered valid and which testimonies of patients’ merit attention or dismissal (Carel and Kidd 2014; Kidd and Carel 2017). In the absence of clear biological markers, chronic pain often remains misunderstood or insufficiently conceptualized, reflecting a hermeneutical injustice in which the medical system lacks the necessary “language” to capture and understand the complexity of patients’ experiences (Carel and Kidd 2014; Tosas 2021). As a result, CPP voices may be marginalized, their suffering unacknowledged, undermining the potential for meaningful, person-centred care.

Hookway’s (2010) concept of “participatory injustice” expands on Fricker’s original framework by showing how unjust exclusion from discussion, deliberation, or inquiry prevents individuals from acting as genuine co-inquirers. This goes beyond Fricker’s focus on the harm done to people as knowledge contributors by underscoring the importance of active participation in the inquiry process. Such exclusion not only constitutes an epistemic harm but also clashes with the core principles of person-centred care, which demand integrating patients as active participants into clinical discussions. Along those lines, Sakakibara (2023) warns that an overreliance on objective findings can reduce patients to “sources of information,” neglecting their crucial role as “informants” who provide indispensable first-hand insights for co-constructing meaningful care. Equally, Dotson’s (2011) notion of “testimonial smothering” shows how prejudiced or dismissive environments can pressure individuals to alter or withhold their testimony, further undermining their capacity to be co-inquirers. While an objectivity-focused approach may be warranted in acute or emergency settings—where measurable indicators guide urgent interventions—it may be insufficient in chronic pain contexts, where subjective accounts seem vital for developing effective management strategies.

We argue that addressing epistemic injustices in chronic pain care necessitates recognizing CPP as active, knowledgeable collaborators in clinical encounters, as demonstrated by our findings. Dell’Olio et al. (2023) argue that genuine person-centred care demands rethinking how patient insights are integrated, emphasizing interactive, reciprocal engagement and the co-creation of knowledge. Our study adds that effective knowledge exchange occurs when CPP feel that HCP acknowledge their own limitations, validate CPP experiences, and engage openly with them as key contributors to their care. We hypothesize such inclusive approach enables CPP to participate in clinical discussions and decisions about how to best manage their condition in real life and helps mitigate testimonial smothering by fostering an open space for sharing experiences without fear of dismissal.

Our findings suggest that when participants felt that they were seen as having medical expertise, it fostered more equitable and participatory clinical relationships. Furthermore, shared cultural or linguistic backgrounds—conceptualized by Bourdieu (2002) as a shared “habitus”—subtly shape social norms and communicative behaviours. This shared habitus may enhance mutual understanding and respect (Dubbin et al. 2013; Medina 2011; Wardrope 2015). By moving beyond a purely objective model and recognizing clinical care as a social practice, HCP may adopt a more reflective and collaborative ethos, addressing the multifaceted nature of chronic pain in a more person-centred manner. To ensure truly person-centred care, HCP must remain mindful of individual differences in culture, language, and expertise, validate patients’ lived experiences, and actively involve them in decision-making. This approach has the potential to reduce epistemic injustices and foster more equitable, effective patient–provider relationships.

Despite growing scholarly recognition of experiential knowledge (Gross and Gagnayre 2017), our findings indicate that the healthcare system still struggles to fully incorporate patient perspectives (Dumez and L’Espérance 2024; Naldemirci et al. (2021), also in the context of chronic pain. This shortfall has significant implications for implementing person-centred care (Dell’Olio et al. 2023; Rogers et al. 2005; Smith et al. 2022), as current conceptualizations often overlook entrenched power imbalances, ethical concerns, and systemic limitations (Entwistle et al. 2018). Notably, only a small fraction of studies on person-centredness include patient voices (Sturgiss et al. 2022), signalling that patient experiences—and the structural factors shaping them—remain insufficiently addressed. Merely shifting from a paternalistic to a patient-centred model risks perpetuating injustices if power differentials are left unexamined (Smith et al. 2022).

## Limitations and Strengths

To bolster data credibility, systematic reflection using a field diary and analytic notes was employed. Overall reflexivity was key to the analytic process as both medical and social science positions were made explicit, discussed, and worked out between the authors.

Although our findings show congruence with literature on CPP experiences with care interactions (MacNeela et al. 2015; Nicola et al. 2021; Roche and Harmon 2017), they provide in-depth insights into the Swiss context specifically. As we did not focus on a specific group CPP, additional research employing intersectional approaches is needed to systematically identify and address the unique challenges faced by particularly marginalized groups. Due to the qualitative study design generalization of the data to other contexts and illnesses is not given. The necessity of online interviews during the COVID-19 pandemic could have biased the data, as not all respondents appreciated the digital format for sharing their personal experiences. Yet, others preferred online interviews due to its convenience—also when face-to-face interviews were again allowed. This resonates with studies showing that online interviews may be preferred by some participants (Hanna 2012), while the quality is comparable to face-to-face interviews (Krouwel et al. 2019).

## Conclusions

Our findings underscore the need to reassess and broaden person-centred care to incorporate the perspectives and expertise of CPP more fully. We show how participants felt dismissed, misunderstood, or relegated to passive roles, revealing a healthcare system that privileges objective data over subjective experience, thereby perpetuating epistemic injustice and limiting opportunities for meaningful, empathetic person-centred care.

A redefined approach to person-centred care should explicitly strive for epistemic reciprocity—seeing patients not merely as informants but as active partners in care, fostering mutual respect and dismantling systemic power imbalances (Dell’Olio et al. 2023; Toro and Martiny 2020). In practice, this means actively involving patients as equal partners in shaping and executing treatment strategies, acknowledging their insights, and systematically integrating their perspectives into research and policy. By adopting these measures, healthcare systems may become more inclusive and equitable, ultimately reducing epistemic injustices, and delivering genuinely person-centred care.

## Data Availability

The datasets generated and analysed in this study are not publicly available owing to the confidentiality and sensitivity of the information provided in the interviews. Anonymized datasets may be available from the corresponding author upon reasonable request.
